# Research on a rapid detection method for systemic lupus erythematosus based on Raman spectroscopy

**DOI:** 10.3389/fimmu.2026.1806438

**Published:** 2026-08-18

**Authors:** Jingbao Rao, Yu Lin, Xiaozhen Zhong, Zhimin Huang, Dan Xiong

**Affiliations:** 1 Shantou University Medical College, Shantou, Guangdong, China; 2 Clinical Laboratory, Shenzhen People's Hospital, Shenzhen, Guangdong, China; 3 Department of Laboratory Medicine, Peking University Shenzhen Hospital, Shenzhen, Guangdong, China; 4 Medical Laboratory, Luohu People’s Hospital, Shenzhen, Guangdong, China; 5 Women and Children’s Health Care Hospital of Luohu, Shenzhen, Guangdong, China

**Keywords:** autoimmune disease (AD), machine learning, peripheral blood mononuclear cell, Raman spectroscopy, systemic lupus erythematosus (SLE)

## Abstract

**Background:**

Systemic lupus erythematosus (SLE) is a multisystem autoimmune disease. Clinical diagnosis is hampered by subjective assessment tools, the low sensitivity and frequent cross-reactivity of traditional biomarkers, and by a limited ability to reflect disease status in real time; moreover, SLE presents phenotypes similar to those of other autoimmune conditions such as RA. There is an urgent need for non-invasive, efficient technologies to improve the accuracy of early diagnosis.

**Methods:**

This study established a rapid method for detecting systemic lupus erythematosus (SLE) using Raman spectroscopy. Raman spectra of peripheral blood mononuclear cells (PBMCs) were obtained from 28 SLE patients and 69 controls, the latter group comprising individuals with rheumatoid arthritis, Sjögren’s syndrome, dermatomyositis, and healthy subjects. After preprocessing the raw spectra, the data were input to several classification models. Finally, a multilayer perceptron (MLP) integrated the feature outputs from each classifier to build an ensemble learner that produced the classification and prediction results.

**Results:**

The LDA results indicated that SLE samples were highly dispersed, reflecting disease heterogeneity, whereas disease control samples formed a tight cluster, indicating molecular homogeneity. In PBMCs from SLE patients, Raman peak intensities for monosaccharides and phenylalanine were higher than in the healthy control group, while the reverse was true for guanine and nucleic acids. Some metabolite peak intensities were higher in the disease comparator group. The integrated model showed the best performance, achieving an accuracy of 93% and an AUC of 0.98; it outperformed single models and effectively discriminated between SLE and control populations.

**Conclusion:**

This proof-of-concept study demonstrates the feasibility of using PBMC Raman spectroscopy with ensemble learning to discriminate SLE from non-SLE populations (including healthy controls and other autoimmune diseases). The observed spectral differences provide descriptive molecular fingerprints that may guide future hypothesis-driven investigations.

## 1 Introduction

Systemic lupus erythematosus (SLE) is a multisystem autoimmune disease characterised by autoantibodies against nuclear antigens, immune complex deposition and chronic inflammation of classic target organs such as the skin, joints and kidneys. The disease course is variable, and persistent inflammation can cause cumulative organ damage and, in severe cases, serious health consequences and premature mortality (1). The pathogenesis involves overactivation of B lymphocytes with production of various autoantibodies, in which abnormal activation of T lymphocytes contributes, resulting in immune hyperactivity and injury to multiple organ systems. Although long-term outcomes for patients with SLE have improved in recent years with advances in medical care, irreversible chronic organ and tissue damage caused by the disease remains a major challenge. Currently, diagnosis of SLE follows the classification criteria issued by the European League Against Rheumatism (EULAR) in 2019 (2). The clinical evaluation of SLE integrates multidimensional data — including clinical symptoms, physical signs, laboratory parameters, organ function and physician assessment — yet it continues to confront three core challenges. First, the choice of assessment tools varies subjectively and is readily influenced by clinicians’ experience, which can lead to missed or delayed diagnoses at early stages. Second, although anti-dsDNA and anti-Sm antibodies are highly specific, the sensitivity of anti-Sm is only about 30% and these antibodies can cross-react with other connective tissue diseases. Finally, conventional biomarkers poorly reflect real-time changes in immune pathology and the progression of chronic damage, producing delayed evaluations that impede timely treatment. These limitations underscore the urgent need for non-invasive, efficient diagnostic technologies to improve the accuracy of early diagnosis.

Rheumatoid arthritis (RA), systemic lupus erythematosus (SLE), Sjögren’s syndrome (SS) and dermatomyositis (DM) are autoimmune diseases (ADs) that are commonly seen in women (3). Although each disorder typically targets particular organs, they share clinical manifestations, serological profiles and immunological features (4, 5). Peripheral blood is central to the immune responses in these ADs, serving as the principal route for immune-cell trafficking and signal transmission in patients with RA, SLE, SS and DM. Peripheral blood mononuclear cells (PBMCs), as the predominant immune cells in peripheral blood, provide a critical link in the initiation of autoimmune inflammatory processes directed at target organs. Consequently, analysing PBMC expression profiles can reveal molecular characteristics of immune cells within affected organs, offering important insights into the immunopathological mechanisms of these diseases (6).

Raman spectroscopy is a scattering technique that exploits molecular vibrational modes to enable non-destructive detection of biomolecules such as proteins, enzymes and nucleic acids. A sample’s intrinsic vibrational frequencies provide a distinctive phenotypic “fingerprint” and reflect its physiological and biochemical state (7–9). Capitalising on the advantages of non-destructive testing and rapid analysis, the integration of Raman spectroscopy with artificial intelligence (AI) for medical diagnostics has accelerated, and its translational potential and practical value have become increasingly apparent. Concurrently, numerous studies have shown that machine-learning models perform well in tasks such as precise classification and early supportive diagnosis of autoimmune diseases, thereby offering important technical support for optimising treatment pathways. Xu J et al. proposed a diagnostic application of Raman spectroscopy in myalgic encephalomyelitis/chronic fatigue syndrome (ME/CFS) diagnosis (10). Chang C et al. proposed a diagnostic application of Raman spectroscopy in SLE diagnosis (11). MI Y et al. proposed a diagnostic application of Raman spectroscopy in neuropsychiatric systemic lupus erythematosus (NPSLE) diagnosis (12, 13). Raman spectroscopy combined with artificial intelligence has proven to be non-destructive and highly specific for diagnosing autoimmune diseases, and there is an urgent clinical need for early, precise screening and stratified diagnosis of SLE. Although many Raman-based diagnostic studies utilize serum or plasma due to their relatively simple matrix and lower intracellular interference, we chose peripheral blood mononuclear cells (PBMCs) as the target sample for three reasons. First, PBMCs are the principal effector cells in the autoimmune cascade of SLE, directly reflecting disease-related immune dysregulation, whereas plasma contains heterogeneous metabolites derived from multiple organs that may dilute or obscure SLE-specific signals. Second, by focusing on PBMCs, we obtain a cell-autonomous molecular fingerprint that is mechanistically closer to the underlying immunopathology. Third, PBMCs are routinely isolated in clinical laboratories, making the approach potentially translatable. We acknowledge that PBMCs have a higher intracellular “density load” – i.e., abundant proteins, nucleic acids, and organelles – which can cause spectral congestion and peak overlap. To mitigate this issue, we employed high-resolution confocal Raman microscopy with a 350 nm excitation spot and averaged 100 acquisitions per cell. This strategy reduces shot noise and improves signal-to-noise ratio, enabling clear separation of overlapping bands. Plasma-based Raman spectroscopy is complementary to our approach and will be explored in future studies. Given the limitations of current diagnostic models, this study developed an integrated diagnostic system based on Raman molecular fingerprinting and multimodal machine-learning algorithms. The aim was to overcome the low efficiency and inadequate specificity of traditional methods, to deliver a practical, operable tool for clinical use, and to accelerate the clinical translation and large-scale application of Raman–AI technology.

While healthy vs. diseased classification can be achieved by simple methods (e.g., SIMCA), the primary clinical challenge is discriminating SLE from other autoimmune diseases with overlapping phenotypes. This requires capturing subtle spectral differences that simple linear models may miss.

## 2 Materials and methods

### 2.1 Study population and sample preparation

The study samples were obtained from the Peking University Shenzhen Hospital Biobank, with approval from the hospital’s Scientific Ethics Review Committee (Approval No. PKU-Shenzhen Medical Ethics Review [2025] No. 100). A total of 97 participants were included in the study: 28 patients with SLE; 39 patients with other autoimmune disorders (24 RA, 9 SS, 6 DM) were included as a disease comparator group (hereafter referred to as RA+SS+DM); and 30 healthy individuals as controls.

The study established clear inclusion criteria. SLE patients had to meet the 2019 International Collaborative Group on Lupus Research (SLICC) classification standards, requiring ≥4 clinical and immunological criteria. The disease comparator group comprised RA patients meeting the 2010 American College of Rheumatology (ACR)/European League of Associations for Rheumatology (EULAR) classification criteria. DM patients had to adhere to the 2017 EULAR/ACR classification standards, while SS patients had to meet the 2016 ACR/EULAR criteria (14–17). Healthy controls were required to show no autoimmune disease-related symptoms or abnormal laboratory indicators. Participants had to be aged between 18 and 70 years (with no gender restrictions), have no other autoimmune diseases or severe complications (e.g., severe infections, malignancies), and all subjects had to provide informed consent. These inclusion criteria ensured sample homogeneity and supported the reliability of the research results.

The exclusion criteria for this study are as follows: Patients with severe organ dysfunction or malignant tumours are excluded due to the potential for nonspecific interference with Raman spectroscopy signals. Pregnant or breastfeeding women are also excluded, based on considerations for foetal and infant safety. Individuals with incomplete diagnostic information, such as lacking comprehensive medical histories or necessary laboratory test results, are excluded if their disease diagnosis cannot be confirmed. Additionally, those with comorbid mental disorders or cognitive impairments who are unable to comprehend the study content or sign informed consent forms are excluded. Strict adherence to these criteria minimises confounding factors and ensures the accuracy and reliability of the research findings.

All blood samples were collected freshly through peripheral venipuncture, utilising EDTA as the anticoagulant. The sample processing workflow involved several steps. First, PBMCs were extracted from the EDTA-treated whole blood using density gradient centrifugation. Subsequently, the isolated PBMCs were resuspended in phosphate buffer solution (PBS) and briefly stored at 4 °C in preparation for subsequent Raman spectroscopy analysis. Prior to Raman acquisition, PBS was removed by deionized water washing as detailed in section 2.2.

### 2.2 Data collection and spectral analysis

The Raman spectroscopy system utilises a WITec alpha 300R coupled with a Raman confocal microscope from Ulm, Germany, consisting of a core host unit and essential supporting components. These components comprise a UHTS 300-type piezoelectric stage from WITec GmbH, a 100× air objective lens with a numerical aperture of 0.90 (Zeiss EC EPIPLAN), a 532 nm green solid-state excitation laser with an output power of 32 mW (WITec GmbH), a Newton-type imaging spectrometer with a 600 groove/mm grating and a -60°C thermoelectric-cooled CCD (Andor Technology Ltd., UK), and a 10μm diameter single-mode quartz fibre optic cable connecting the spectrometer and objective lens. The core detection parameters include a laser excitation spot size of 350 nm, a wavelength acquisition range of 0-3,600 cm^-1^, constant laser intensity during collection, an integration time of 0.1s, and an 8 μm×8 μm fast scanning area.

Spectral data were then acquired from each spot as described below: before spectral acquisition, PBMC samples stored in PBS were centrifuged (1500 rpm, 5 min) to pellet the cells. The supernatant was discarded, and the pellet was washed twice with 500 µL deionized water (1500 rpm, 10 min each) to remove residual PBS salts, which can produce strong Raman interference (e.g., phosphate vibrations near 940 cm^-1^). After the final wash, the pellet was resuspended in 20–30 µL deionized water. Take 2 μL of the homogenized PBMC-Raman suspension and transfer it onto a aluminium slide (Brand: Deuterium Peak D-Band, Model: NO.1001). Allow to dry at room temperature before performing the detection on the instrument.

For each sample, Raman spectra were acquired from approximately 50 individual PBMCs. The spectrum from each cell was collected independently (100 accumulations per cell) (18). To obtain a single representative spectrum per sample, the 50 single-cell spectra were averaged. This averaged spectrum was then used for subsequent preprocessing and model analysis. Therefore, although measurements were performed at single-cell resolution, the final dataset represents bulk population-level molecular fingerprints of PBMCs. Machine learning algorithms like Support Vector Machines (SVM) and Random Forest were used to develop diagnostic models, assessing their ability to identify SLE. Cross-validation was performed to validate the models’ accuracy and reliability. The classification outcomes demonstrated the promise of Raman spectroscopy in swiftly detecting SLE. Subsequently, the detection approach underwent validation in clinical environments to confirm its practical utility.

The study adhered to standard procedures for collecting and analysing Raman spectroscopy data. The steps included selecting 50 cells from each PBMC-Raman sample for data collection, using a 6×6-point sampling pattern. Subsequently, the average spectra of all sampled points per cell were computed to mitigate individual point variations. The spectral analysis involved three main stages: screening abnormal spectra by applying signal quality thresholds to eliminate noise, sequentially removing interference signals (cosmic ray contamination, fluorescence baseline drift, and residual noise) to enhance signal-to-noise ratio and spectral purity, and normalising areas to standardise spectral integration across samples, reducing interference from variations in spectral intensity. This method established a consistent data basis for subsequent spectral feature extraction and model analysis.

**Figure 1:**
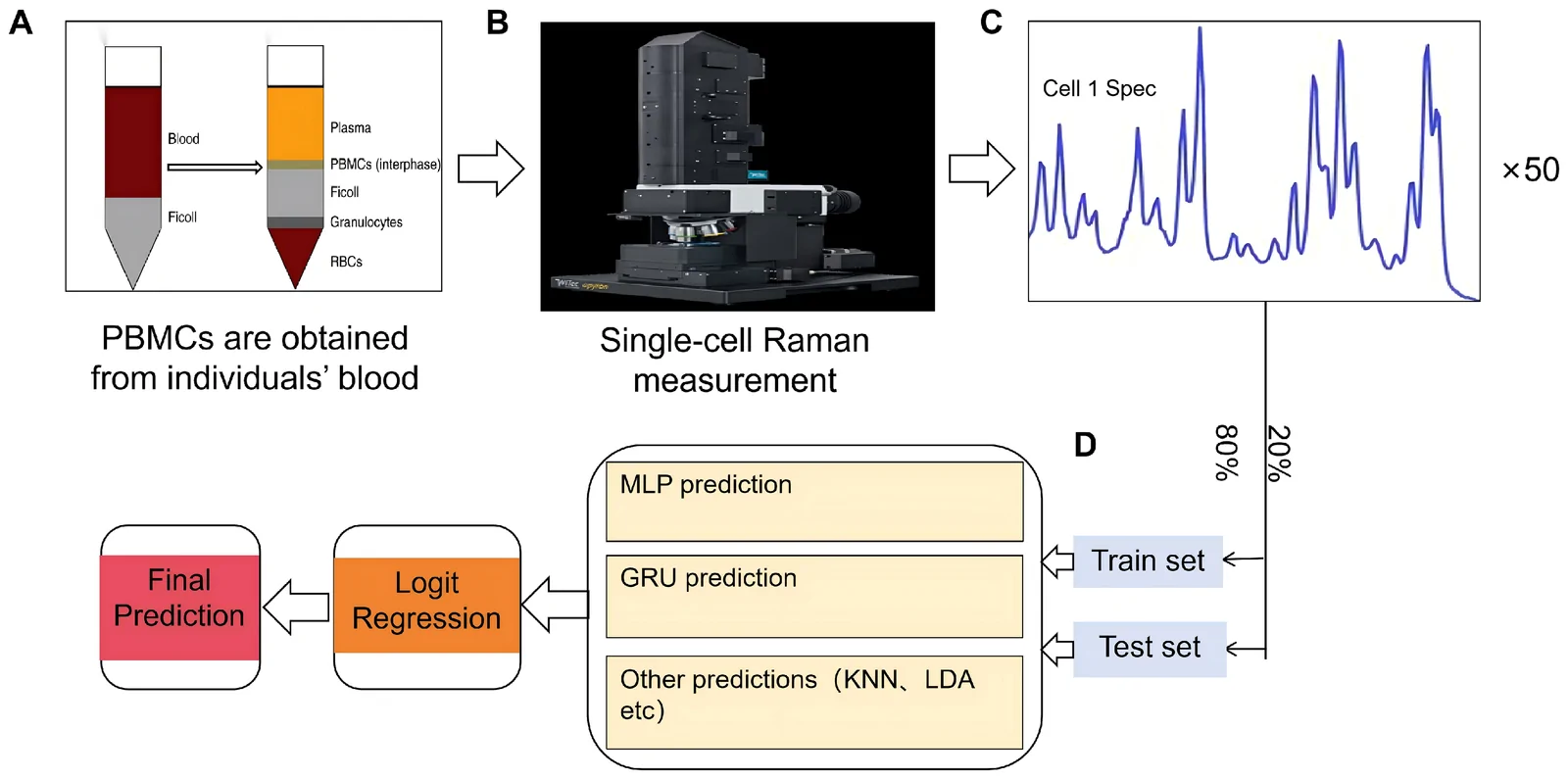
Schematic diagram of the population-level SLE blood Raman spectroscopy diagnostic test. **(A)** Isolation of PBMCs from blood samples. **(B)** Raman spectroscopy measurements of individual PBMCs from 97 individuals. **(C)** Approximately 50 spectra were obtained per cell. **(D)** The PBMC-Raman data from 97 individuals were divided into a training set (80%) and a test set (20%) with balanced subgroup distribution. The training set was employed to train an ensemble learner, while the independent test set was utilised to evaluate the trained model, diagnosing cells as healthy controls (HC), SLE patients, or disease comparator groups (SS+RA+DM).

The Raman spectroscopy data modelling process adheres to a standardised workflow: “Data Segmentation - Preprocessing - Model Learning - Result Integration”. Initially, the data is segmented by randomly partitioning the subjects (e.g., patients with systemic lupus erythematosus, disease comparator groups, and healthy control groups). Subsequently, 80% of the participants from each category are randomly chosen as the training set to construct a model database, with the remaining 20% assigned to the test set (19). To ensure the impartiality and dependability of the results and prevent data leakage from influencing the evaluation, a blind test approach is employed to validate the model’s performance. To enhance the model’s discriminative capacity, a machine learning technique that combines multiple classification algorithms is utilised, demonstrating superior performance compared to employing individual algorithms. In this investigation, the PBMC-Raman is applied to 13 distinct classification models, including MLP, ANN, GRU, KNN, LDA, among others. Following this, a dedicated MLP is employed to consolidate the outputs from various classifiers, establishing an ensemble learner to deliver the final diagnosis (**Figure 1**). To exclude potential spectral interference from the sample preparation procedure, we analysed solvent blanks (2 μL deionized water directly on an aluminium slide) and process blanks (500 μL deionized water carried through the same centrifugation, washing, and resuspension steps as the PBMC samples). Both blanks yielded negligible Raman signals (<5% of sample mean intensity) with no characteristic peaks in the 500–1800 cm^-1^ region (**Supplementary Figure 2**). These results confirm that the observed spectral features originate from the PBMCs themselves and not from residual EDTA, PBS, deionized water impurities, or the aluminium substrate.

Peripheral blood mononuclear cells (PBMCs) play a central role in both innate and adaptive immunity by dynamically modulating immune cell activation and differentiation processes through their biochemical composition, encompassing proteins, nucleic acids, lipids, and carbohydrates. This active involvement positions PBMCs to contribute directly to the pathological dysregulation observed in rheumatic autoimmune diseases. Raman spectroscopy, a label-free vibrational analysis method relying on non-elastic light scattering, allows for the non-invasive detection of cellular biomolecules by capturing distinct molecular vibration patterns (Raman peaks) linked to specific functional groups or biomolecular categories. This study aims to create a molecular fingerprint profile of PBMCs to lay the groundwork for understanding immune-mediated biochemical alterations in rheumatic diseases by systematically identifying and characterising the principal Raman peaks in PBMCs (20). The key findings are outlined in **Table 1**.

**Table 1:** Peak location and distribution of main Raman bands in PBMCs.

Wavenumber (cm^-1^)	Assignment
749	Symmetric breathing of tryptophan (protein assignment)
782	DNA
856	Amino acid side chain vibrations of proline
898	Saccharide band
1002	Phenylalanine
1086	n(C-C) gauche
1204	Amide III
1233	Amide III (arising from coupling of C-N stretching)
1366	Guanine, TRP (protein)
1447	Lipids
1455	Deoxyribose
1550	Methylene deformation in biomolecules
1603	phenylalanine (protein assignment)
3060	Unsaturated fatty acids

This study evaluates the classification model’s performance using sensitivity, specificity, and accuracy. The calculation of these indicators relies on four essential outcomes: true positive (TP), true negative (TN), false positive (FP), and false negative (FN). The formulas for calculating sensitivity, specificity, and accuracy are presented in Equations 1–3.

(1) $$Sensitivity=\frac{TP}{TP+FN}\times 100\%$$

(2) $$Specificity=\frac{TN}{TN+FP}\times 100\%$$

(3) $$Accuracy=\frac{TP+TN}{TP+TN+FP+FN}\times 100\%$$

### 2.3 Statistical analysis

Statistical analyses were performed using Python (version 3.9) with the SciPy library. Differences between groups were assessed using two-tailed Student’s t-tests with Bonferroni correction for multiple comparisons. In this study, each data point represents an individual PBMC at the single-cell level. A P-value < 0.05 was considered statistically significance. Significance levels are indicated as: ns, P ≥ 0.05; *, P < 0.05; **, P < 0.01; ***, P < 0.001; ****, P < 0.0001.

## 3 Results

### 3.1 Study population and clinical characteristics

Our preliminary study involving 10 individuals demonstrated that PBMC-RamanRaman spectroscopy, when combined with machine learning methods, can effectively distinguish patients with systemic lupus erythematosus (SLE) from healthy controls (HC). Specific biomarkers were suggested in the peripheral blood mononuclear cells (PBMCs) of SLE patients. To further investigate the potential of this technology for SLE diagnosis, we increased the sample size and included a disease comparator group exhibiting similar clinical symptoms, comprising individuals with rheumatoid arthritis (RA), Sjögren’s syndrome (SS), and dermatomyositis (DM). This development aimed to establish a blood-based diagnostic test using PBMC-RamanRaman spectroscopy. **Table 2** summarises the main characteristics of the SLE cohort, the disease comparator group (RA + SS + DM), and the healthy control group in this study. A total of 97 participants were included, comprising 28 SLE patients, 30 healthy controls, and 39 disease controls (RA, n=24; SS, n=9; DM, n=6).

**Table 2:** Comparison of clinical and laboratory indicators between autoimmune disease groups and healthy control groups.

Characteristic	SLE (n=28)	RA (n=24)	SS (n=9)	DM (n=6)	HC (n=30)
*Age (years)	40.7±12.0	51.7±10.4	52.9±14.8	39.3±7.9	42.0±12.8
Gender [n (%)]					
Female	28 (100)	22 (91.7)	9 (100.0)	3 (50.0)	17 (56.7)
Male	0 (0.0)	2 (8.3)	0 (0.0)	3 (50.0)	13 (43.3)
^+^ESR (mm/h)	16.0 (10.0,33.0)	33.0 (12.0,51.0)	20.5 (13.5,40.0)	14.0 (6.8,35.3)	10 (2,15)
PLT (×10^9^/L)	223±73.0	280.7±151.2	208.1±71.9	240.8±77.8	255.8±48.3
ANA [n (%)]					
Negative	2 (7.1)	5 (20.8)	9 (100.0)	2 (33.3)	30 (100)
Positive (titer ≥1:100)	26 (92.9)	19 (79.2)	0 (0.0)	4 (66.7)	0 (0.0)
Anti-dsDNA [n (%)]					
Negative	20 (71.4)	23 (95.8)	8 (88.9)	6 (100.0)	30 (100)
Positive (≥50 IU/mL)	8 (28.6)	1 (4.2)	1 (11.1)	0 (0.0)	0 (0.0)
Clinical symptoms [n (%)]					
Skin rash	8 (28.6)	1 (4.2)	3 (33.3)	4 (66.7)	0 (0.0)

*Mean±SD、+Median (Quartile range) or n (%) (discrete variables),the ESR is expressed as a median (quartile). SLE, Systemic Lupus Erythematosus; RA, Rheumatoid Arthritis; SS, Sjögren's Syndrome; DM, Dermatomyositis; HC, Healthy Control; ESR, erythrocyte sedimentation rate; PLT, platelet; ANA, antinuclear antibody.

### 3.2 PBMC-Raman differentiate the different queues

**Figure 2:**
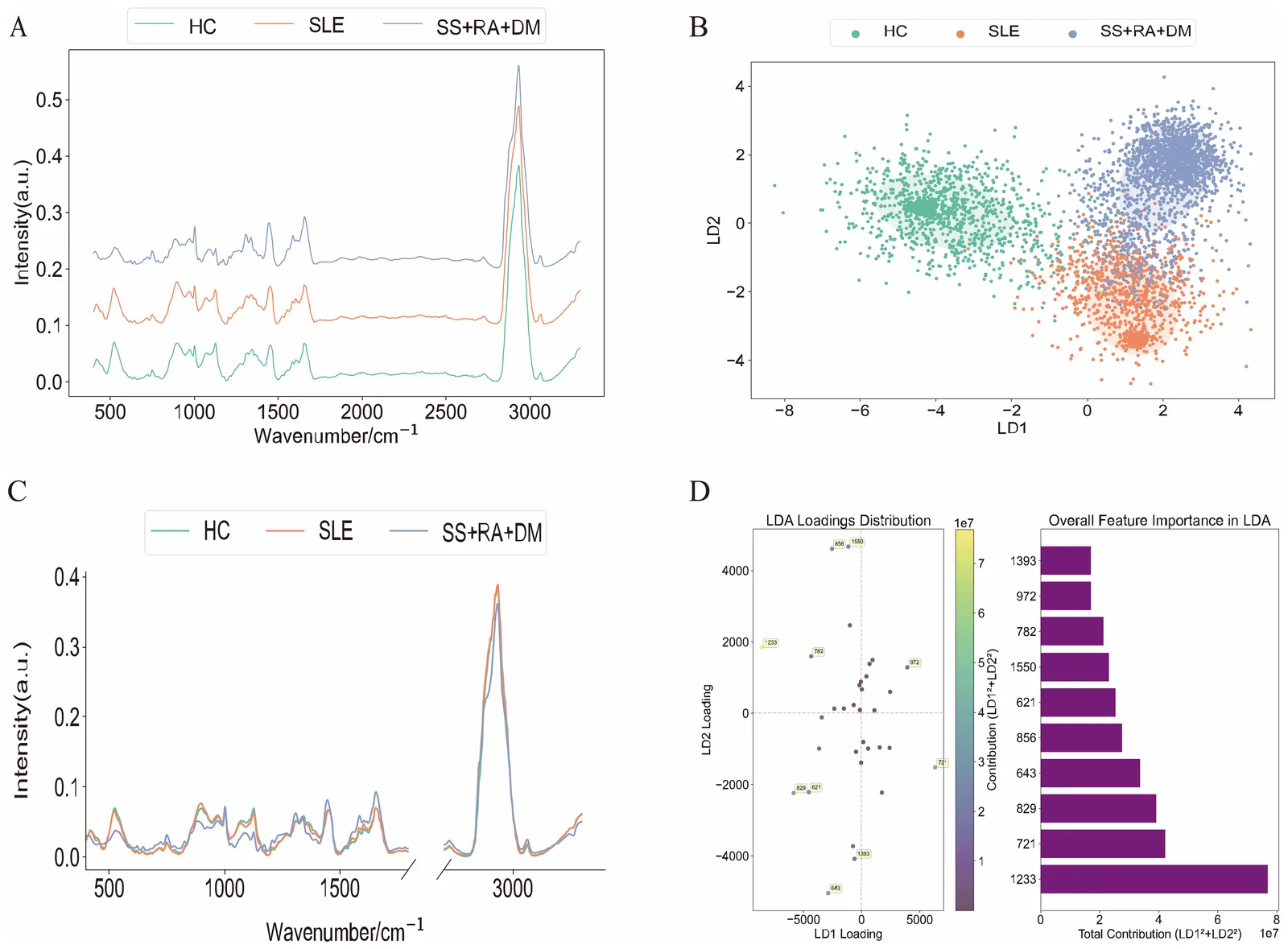
Raman spectroscopy and dimensionality-reduction analysis of cohorts (n=97). **(A)** Overlaid raw Raman spectra for healthy controls (HC, green), systemic lupus erythematosus (SLE, orange) and a composite group (SS+RA+DM, blue) across the full wavenumber range (500–3200 cm^-1^). **(B)** LDA projection onto the first two discriminant axes (LD1, LD2), showing group separation driven by spectral features (green = HC, orange = SLE, blue = RA+SS+DM). **(C)** Expanded view of the fingerprint region (500–1800 cm^-1^), emphasising subtle structural differences. **(D)** LDA feature loadings with overall feature importance.

In this study, PBMC-Raman was used to analyse PBMCs from 97 samples, yielding 4,634 spectra. These comprised 1,287 spectra from patients with SLE, 1,431 from HC, and 1,916 from the RA + SS + DM group. All Raman measurements were performed blind. **Figure 2A** shows the Raman of individual PBMCs in the fingerprint region (500–3,200 cm^-1^). This region contains most of the information on intrinsic molecular vibrations within cells and can therefore be regarded as the cell’s “fingerprint”.

Supervised linear discriminant analysis (LDA) was applied to reduce the high dimensionality and noise of the Raman spectra. The LDA plot of all PBMC-Raman spectra along LD1 and LD2 revealed three distinct clusters corresponding to SLE, HC, and RA+SS+DM respectively (**Figure 2B**). The wide scatter of SLE samples reflects the disease’s high heterogeneity, i.e. diversity of phenotypes and molecular mechanisms, whereas the tight clustering of RA+SS+DM samples indicates highly homogeneous molecular characteristics, suggesting shared abnormalities in biological pathways.

In **Figure 2C**, within the fingerprint region (500–1,800 cm^-1^, which primarily corresponds to vibrational modes of amino acid side chains, nucleic acid bases and carbohydrates), subtle differences in characteristic peaks between groups are evident. At approximately 898 cm^-1^ (saccharide band, corresponding to carbohydrate vibrations), the peak for the RA + SS + DM group shifted markedly left to 886 cm^-1^ relative to the HC group and SLE patients. At 1455 cm^-1^ (deoxyribose, corresponding to deoxyribose vibrations), the RA + SS + DM group also showed a leftward shift to 1447 cm^-1^, yielding a clear distinction from the HC and SLE peak positions. A leftward shift of a peak generally reflects changes in the chemical structure or conformation of the associated biomolecule. These observations indicate that, in PBMCs from the disease comparator group, carbohydrates and deoxyribose may share molecular modifications or metabolic remodelling — corroborating the earlier LDA finding of “highly homogeneous molecular characteristics” in the disease comparator group. They further confirm that, although SS, RA and DM are distinct disease, they share common alterations in the vibrational properties of core biomolecules. This contrast in characteristic peak shifts with the stable peak positions observed in the HC and SLE groups provides key spectral markers for building discriminative models and supplies direct molecular phenotypic evidence to explain why the integrated model could efficiently distinguish SLE from other rheumatic immune diseases.

The left panel of **Figure 2D** shows the load distribution of LD1 and LD2 across wavenumbers, while the right panel displays the overall feature-importance ranking. In the load plot, specific wavenumbers (for example, 1233, 721 and 829 cm^-1^) have substantial loadings on LD1 or LD2 and therefore drive the separation between groups. The bar chart on the right shows that 1233 cm^-1^ makes the largest contribution overall, followed by 721 and 829 cm^-1^. These wavenumbers correspond to characteristic vibrational modes of biomolecules: 1233 cm^-1^ relates to protein amide III bands, and 829 cm^-1^ to nucleic-acid phosphate-backbone vibrations. Abnormalities at these bands indicate alterations in disease-related nucleic acids, proteins and lipids. Accordingly, combining differences in characteristic Raman peaks with dimensionality-reduction clustering and feature-importance analysis reveals disease-associated changes in nucleic acids, proteins and lipids through spectral variation. Moreover, these key wavenumbers provide a basis for investigating disease mechanisms and for developing biomarkers for precise diagnosis.

Raman spectroscopy permits label-free detection of biomolecular alterations by capturing characteristic vibrational patterns of nucleic acids, proteins and lipids. To determine whether Raman spectra distinguish SLE, healthy controls (HC) and other rheumatic diseases (SS + RA + DM), we performed hierarchical clustering on spectral features with differential abundance (online **Supplementary Figure 1**). The resulting clusters clearly separated the three groups: SLE and HC samples showed greater spectral similarity, with more tightly grouped columns, whereas the SS + RA + DM samples formed a more dispersed cluster, indicating distinct molecular signatures relative to SLE and HC. Several spectral features exhibited high intensity (red) in both SLE and HC but low intensity (blue) in SS + RA + DM; these included 1072, 418, 524 and 1393 cm^-1^. Such bands may represent physiological or SLE-associated molecules—for example, glycogen C–O stretching near 1070 cm^-1^ and CH_2_ bending modes related to protein secondary structure near 1390 cm^-1^. By contrast, features at 1338, 782, 1172 and 621 cm^-1^ showed lower intensity in SLE and HC but elevated intensity in SS + RA + DM; these may indicate dysregulated molecules in SS, RA or DM, for instance the cyclic breathing modes of pyrimidine rings in DNA/RNA near 780 cm^-1^ and C–C stretching of lipid acyl chains near 1170 cm^-1^. Notably, characteristic peaks at 2875 and 2934 cm^-1^, corresponding to CH_2_/CH_3_ stretching vibrations in lipids and proteins, were also observed. The samples exhibited heterogeneous intensities in both SLE and HC, indicating subclinical molecular variability even in otherwise healthy individuals. These findings demonstrate that Raman spectroscopy can discriminate molecular heterogeneity among rheumatic diseases; the differential spectral features provide candidate avenues for disease-specific biomarkers, while the clustering patterns suggest a shared-plus-unique pathogenic mechanism between SLE and other rheumatic disorders.

### 3.3 Biomolecular quantification across diverse cohorts

**Figure 3:**
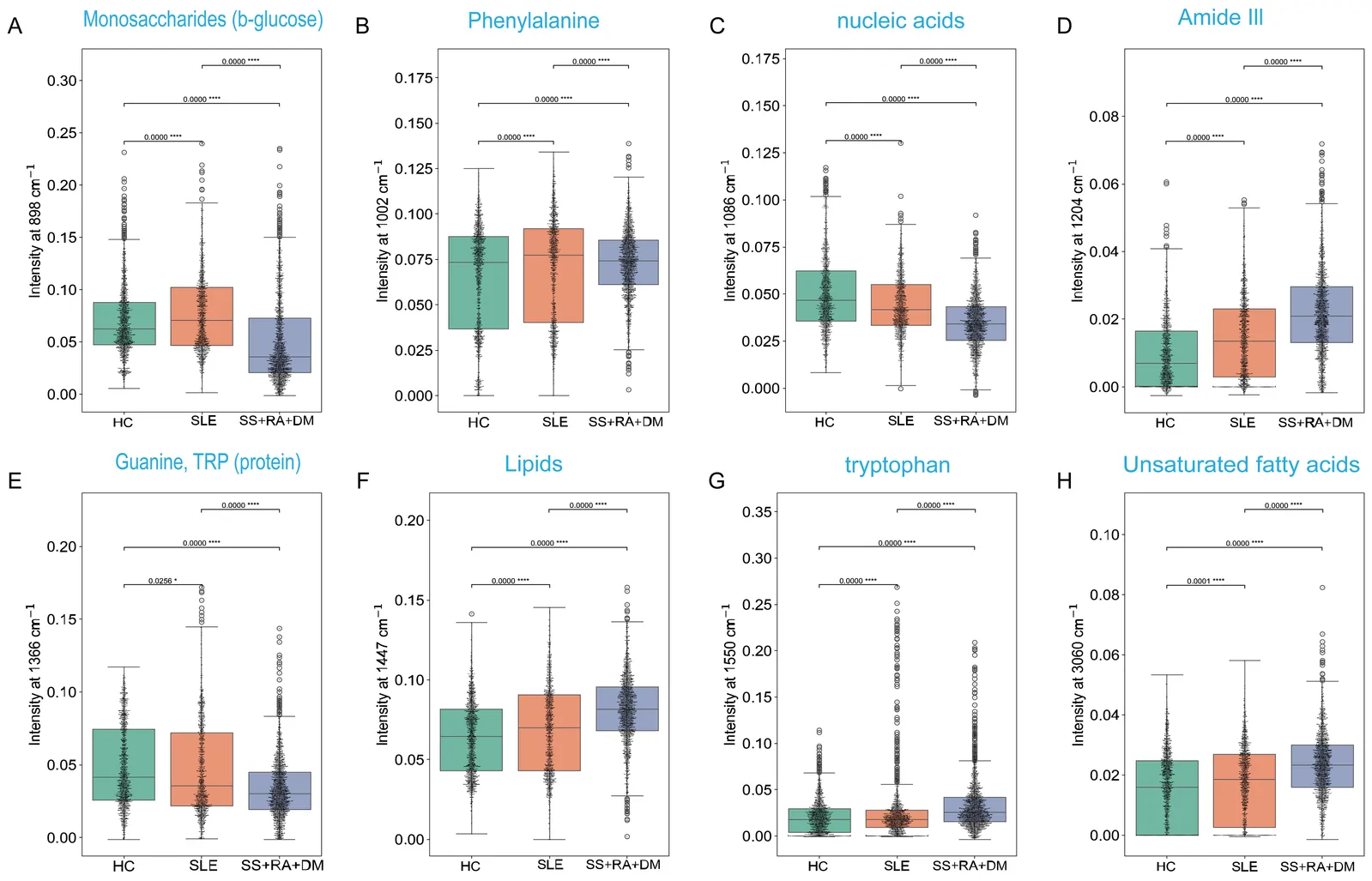
Raman spectral intensities of key biomolecules across study groups. The box plots, overlaid with individual data points, show Raman intensity at selected wavenumbers for three groups: healthy control (HC, green), systemic lupus erythematosus (SLE, orange) and disease control (SS+RA+DM, wathet). The vertical axis denotes Raman intensity and the horizontal axis denotes study groups. Statistical significance was assessed by the Kruskal–Wallis test with Dunn’s multiple comparisons; **** indicates P<0.0001, and exact P-values for specific pairwise comparisons are shown. The biomolecules and their corresponding Raman wavenumbers are: **(A)** monosaccharides (e.g. β-glucose) at 898 cm^-1^; **(B)** phenylalanine at 1002 cm^-1^; **(C)** nucleic acids at 1086 cm^-1^; **(D)** amide III (from C–N stretching coupling) at 1233 cm^-1^; **(E)** guanine and tryptophan (protein-derived moieties) at 1366 cm^-1^; **(F)** lipids at 1447 cm^-1^; G) tryptophan (protein-associated signal) at 1550 cm^-1^; **(H)** unsaturated fatty acids at 3000 cm^-1^.

Using LDA as a feature-selection tool to identify the most significant Raman peaks facilitated group separation. Quantitative analysis of those significant peaks across the HC, SLE and RA+SS+DM groups highlighted differences in cellular biochemistry between healthy individuals and patients (21). In this study, we found that, in the SLE cohort, Raman intensities corresponding to monosaccharides and phenylalanine in PBMCs were higher than in the HC group and the SS+RA+DM group (**Figures 3A, B**). These peaks may serve as potential biomarkers for SLE in PBMCs. By contrast, Raman intensity attributable to nucleic acids was lower in both the SLE and SS+RA+DM groups than in the HC group (**Figure 3C**). Notably, in the SS+RA+DM group, Raman intensities for amide III (reflecting protein secondary structure), guanine, lipids, tryptophan and unsaturated fatty acids were higher than in the SLE and HC groups (**Figures 3D–H**). This pattern indicates that elevated levels of these metabolites characterise abnormal PBMC metabolism and that their specificity may relate to the underlying disease composition. Furthermore, all patient groups differed significantly from the HC group. These spectral assignments are based on previously reported biomolecular assignments; they represent relative vibrational signal differences rather than absolute molecular quantification, and require validation by metabolomics or functional assays.

### 3.4 A diagnostic detection technique integrating PBMC-Raman spectroscopy with ensemble learning algorithms

Although LDA has proven effective at separating different disease cohorts, we adopted an ensemble approach to further enhance discriminatory performance. Ensemble learning integrates multiple classifiers to achieve better performance than any single algorithm alone. We supplied the PBMC-Raman to 13 classification models—Multi-Layer Perceptron (MLP), Artificial Neural Network (ANN), Gated Recurrent Unit (GRU), K-Nearest Neighbours (KNN), LDA, Gradient Boosting (GB), Convolutional Neural Network (CNN), Random Forest (RF), Linear Support Vector Machine (LSVM), Quadratic Discriminant Analysis (QDA), Logistic Regression (LR), Radial Basis Function Support Vector Machine (RSVM) and Naive Bayes (NB)—and calculated the predictive performance of each model (**Table 3**). An ensemble learner was then constructed to produce the final diagnosis.

**Table 3:** Single spectral prediction results from various machine learning models.

Model	Accuracy	Sensitivity	Specificity
MLP	0.86	0.85	0.93
ANN	0.85	0.84	0.93
KNN	0.82	0.82	0.91
GB	0.81	0.81	0.91
CNN	0.81	0.8	0.91
RF	0.8	0.8	0.9
GRU	0.8	0.79	0.9
LSVM	0.77	0.77	0.89
QDA	0.76	0.75	0.88
LDA	0.75	0.75	0.88
LR	0.75	0.75	0.88
RSVM	0.69	0.68	0.85
NB	0.64	0.65	0.83

**Figure 4:**
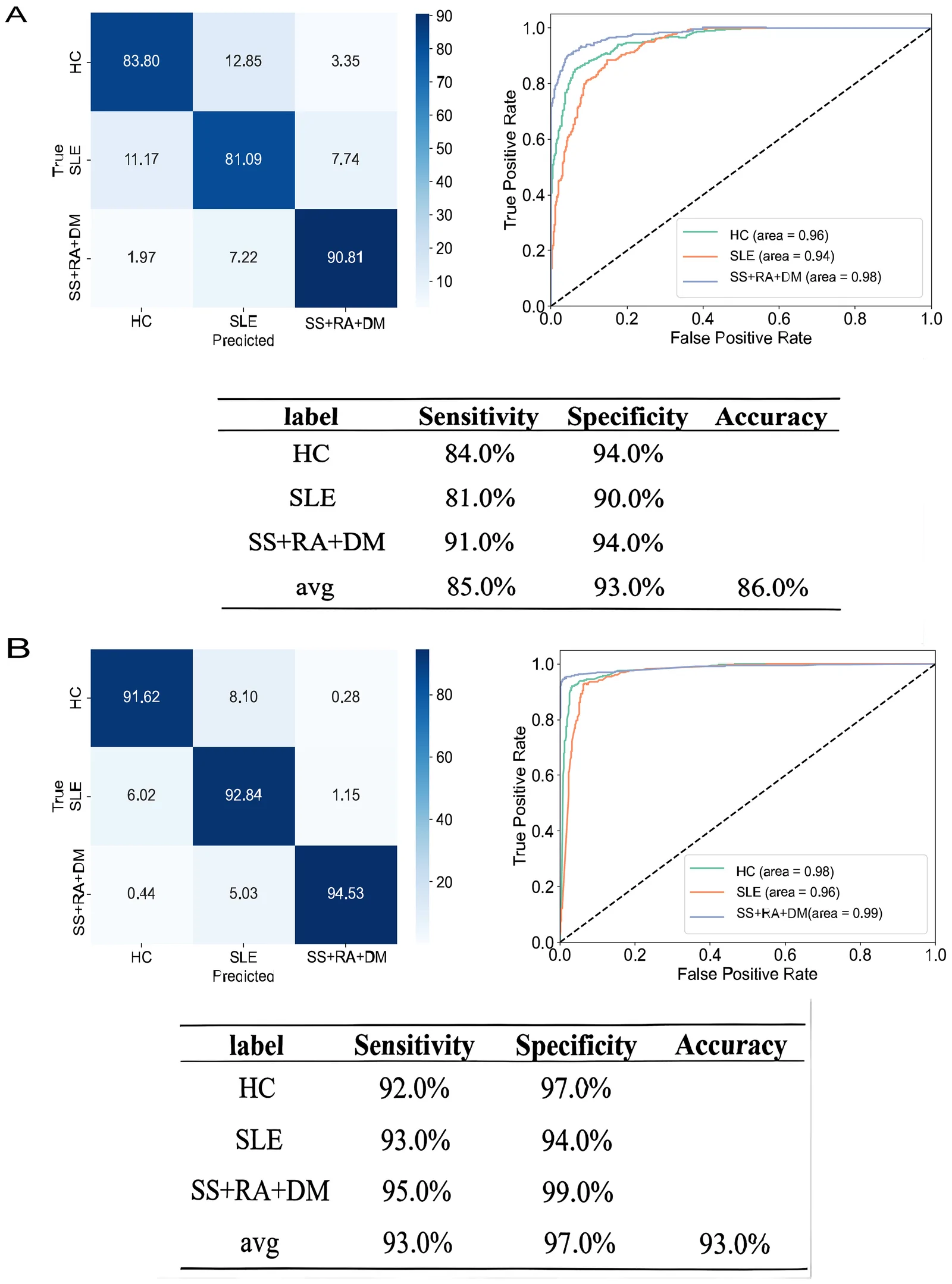
Comparison of discriminative performance of two models across research cohorts. **(A)** Optimal single-model predictions, comprising: a confusion matrix (rows = true labels; columns = predicted labels; values = classification accuracy), an ROC curve (x-axis = false positive rate; y-axis = true positive rate; AUC in the legend), and a table of performance metrics (sensitivity, specificity, accuracy). **(B)** Ensemble model predictions, presented in the same format as A, which improve performance by integrating multi-omics and multimodal data.

The ensemble model’s accuracy rose markedly from 64% in the single-model setting to 93%, indicating that integrating diverse learning algorithms enhanced predictive performance. The ensemble model accurately discriminated HC, SLE, and the combined RA+SS+DM group by disease type. The AUC for SS+RA+DM increased from 0.98 in the single model to 0.99 in the ensemble, while the AUCs for HC and SLE improved from 0.96 and 0.94 to 0.98 and 0.98 respectively, indicating a stronger capacity to separate true positives from false positives. Mean sensitivity, specificity and accuracy increased substantially from 0.85, 0.93 and 0.86 in the single model to 0.93, 0.97 and 0.93 in the ensemble, emphasising the benefit of multi-feature fusion for differentiating complex diseases. By integrating multi-dimensional features, the ensemble model outperformed single-model approaches in distinguishing rheumatologic–immunologic diseases from healthy controls and provided robust support for the development of precision diagnostic tools in this field, particularly by offering higher discriminative value for the clinically complex SS+RA+DM population (**Figures 4A, B**). Notably, the ensemble MLP model provides robust classification but limited inherent interpretability. LDA feature loading and importance analysis were used to identify biologically relevant spectral bands, which serve as the main interpretive basis. Further development of explainable artificial intelligence (XAI) will help directly map Raman shifts to model decision-making.

## 4 Discussion

Diagnosing SLE remains challenging because of its heterogeneous clinical presentations, the low sensitivity of classical biomarkers (for example, anti-Sm antibodies with approximately 30% sensitivity), and the inability of traditional detection methods to capture real-time immune–pathological dynamics (22, 23). This study addresses these unmet needs by developing a non-invasive PBMC-Raman diagnostic platform for SLE based on PBMCs and integrated learning. The study has two principal contributions. First, it achieved high-precision discrimination between SLE, healthy controls (HC), and other rheumatic autoimmune diseases (SS+RA+DM), with a diagnostic accuracy of 93%. Second, it tentatively identified candidate molecular fingerprints that illuminate underlying immune metabolic dysregulation. These findings not only advance SLE diagnostic technologies but also deepen understanding of the disease’s pathobiology. These spectral assignments are based on literature references; independent metabolomic validation (e.g., LC-MS) is required to confirm the identity and concentration of these metabolites.

As listed in **Table 3**, the optimal standalone single model (MLP) yields an accuracy of 0.86, whereas our proposed ensemble learning model improves diagnostic accuracy up to 0.93 across the full dataset. The ensemble framework not only lifts overall classification performance, but also optimizes the identification of rare small-sample autoimmune subtypes, verifying its indispensable value for this diagnostic task. This performance significantly surpassed existing Raman spectroscopy–based diagnostic methods: Chang et al. (11) achieved an accuracy of 88% by applying serum Raman spectroscopy combined with a spiking neural network, with a cohort of 164 participants including 74 SLE patients and 94 healthy controls. In the present study, we obtained a higher accuracy of 93% using peripheral blood mononuclear cells (PBMCs), based on a total of 97 samples comprising 28 SLE cases, 30 healthy controls, and 39 disease comparator subjects. Direct comparison between the two studies is hampered by discrepancies in sample matrices and sample sizes. Importantly, our established PBMC-Raman based on PBMCs exhibited better diagnostic performance. This superiority is presumably due to the fact that PBMCs act as central effector cells that directly participate in the autoimmune cascade underlying SLE pathogenesis (6). Unlike serum, which contains heterogeneous metabolites derived from multiple organs, PBMCs provide cell-autonomous readings of immune dysregulation, enabling more specific disease characterisation.

A principal clinical challenge addressed in this study is the differential diagnosis of SLE from rheumatic conditions with similar presentations, such as SS, RA and DM. Hierarchical clustering and linear discriminant analysis (LDA) revealed distinct clusters with characteristic spectral profiles for SS+RA+DM patients, whereas SLE partially overlapped with both healthy controls (HC) and the SS+RA+DM group. This finding reflects the recognised phenotypic heterogeneity of SLE (24). Accurately distinguishing SLE from other autoimmune diseases is particularly important because misdiagnosis (for example, mistaking SLE for SS) frequently results in inappropriate immunosuppressive treatment and a greater risk of organ damage. The integrated model achieved 95% specificity for SS+RA+DM (**Figure 4B**), thereby reducing the likelihood of misclassifying patients with non-SLE rheumatic disease. This advance addresses a critical gap in current diagnostic pathways.

This study suggested specific biomolecular alterations in PBMCs from SLE patients that correspond with established immunopathological mechanisms of the disease, while also revealing novel candidate biomarkers. Two distinguishing features are particularly noteworthy: increased monosaccharide levels (898 cm^-1^) and elevated phenylalanine (1002 cm^-1^). The increased Raman intensity at 898 cm^-1^ (carbohydrate region) in SLE PBMCs raises the hypothesis that altered glucose metabolism, such as enhanced glycolysis, may be present in SLE immune cells. This is consistent with previous reports of metabolic reprogramming in SLE T cells (25). However, direct evidence (e.g., glucose uptake assays or metabolomics) is required to confirm this hypothesis. Our data demonstrated that PBMC-Raman could detect dysregulation of glucose metabolism in PBMCs, providing a non-invasive window onto immune cell metabolism. Phenylalanine, a precursor of tyrosine important for catecholamine synthesis and T-cell receptor signalling, was elevated in SLE PBMCs. This finding may indicate impaired amino acid catabolism or increased metabolic demand from activated T cells, consistent with Yukai et al.’s observation that PBMCs drive autoimmune activation in SLE (6). By contrast, reductions in guanine/nucleotide-related signals (1366 cm^-1^ and 1086 cm^-1^) were present in both SLE patients and disease controls, suggesting a pan-rheumatic hallmark of nucleic acid dysregulation in immune cells. This observation aligns with the central role of nucleic acid-sensing pathways (for example, TLR7/9) in the pathogenesis of diverse autoimmune diseases (26). Collectively, these spectral signatures not only improve diagnostic specificity but also provide a molecular atlas for SLE, guiding future research into metabolic or immunomodulatory therapies.

Compared with conventional diagnostics, the PBMC-Raman-based approach offers three principal technical advantages. First, unlike invasive biopsies (for example, renal biopsy for lupus nephritis) or antibody-based assays (for example, anti-dsDNA), PBMC-Raman requires only 50 μL of EDTA-coated blood and no chemical labelling, substantially reducing sample preparation time and minimising operator error (7). Second, spectral acquisition (50 cells per sample, averaging 100 measurements per cell) and model analysis are completed within 2 hours, substantially faster than serological assays (for example, anti-Sm antibody testing, which requires 1–2 days) or imaging studies (for example, MRI for organ-damage assessment). Third, conventional biomarkers (for example, complement C4) correlate poorly with acute flares (27); our data show that PBMC-Raman detects dynamic biomolecular changes (for example, lipid and protein conformational shifts) that may better reflect disease activity. Specifically, reduced CH-associated lipid/protein peaks at 3000 cm^-1^ in SLE act as alternative indicators of immune-cell activation and may support more timely treatment adjustments. Together, these characteristics indicate that PBMC-Raman is a promising tool for early SLE screening (for example, in asymptomatic individuals with positive ANA) and for monitoring treatment response (for example, assessing the efficacy of belimumab or JAK inhibitors).

## 5 Conclusions

This proof-of-concept study established a label-free rapid diagnostic strategy based on confocal Raman spectroscopy of peripheral blood mononuclear cells combined with ensemble machine learning for differential diagnosis of systemic lupus erythematosus. Three major core findings were obtained: First, LDA and hierarchical clustering confirmed obvious molecular spectral separation among SLE patients, patients with other rheumatic autoimmune diseases (RA, SS, DM) and healthy subjects. SLE samples presented highly scattered distribution reflecting disease heterogeneity, while the disease comparator group shared consistent spectral features with unified biomolecular metabolic alterations. Second, characteristic peak analysis identified SLE-specific spectral biomarkers: significantly elevated Raman intensities of monosaccharides and phenylalanine in PBMCs from SLE patients, whereas nucleic acid signals decreased universally across all autoimmune cohorts. Third, the integrated ensemble learning model outperformed all single machine learning algorithms, achieving an overall diagnostic accuracy of 93% and an AUC of 0.98, which effectively solved the clinical difficulty of misdistinguishing SLE from phenotypically overlapping autoimmune diseases.

This work verifies the translational potential of PBMC Raman molecular fingerprints as a minimally invasive, rapid auxiliary diagnostic tool for SLE. Compared with traditional serological antibody detection, the proposed method requires only trace peripheral blood, avoids chemical labelling, and can complete sample detection and model prediction within 2 hours. It is expected to support early screening of suspected SLE patients with positive ANA antibodies and real-time monitoring of disease immune activity.

Future work will expand multi-centre cohorts with larger sample sizes, integrate multi-omics data to clarify the causal relationship between altered Raman spectral signals and SLE immune pathogenesis, and develop miniaturized portable Raman detection equipment combined with cloud artificial intelligence models to realize bedside rapid detection of lupus erythematosus in clinical practice.

## 6 Limitations of the study

This study has several inherent limitations, which need to be objectively considered when interpreting the experimental results. At the same time, these deficiencies also point out the direction for improvement for subsequent research.

First, this study is a proof-of-concept trial with a total sample size of only 97 cases. Moreover, the number of cases in the Sjogren’s syndrome and dermatomyositis subgroups is relatively small, and the sample distribution within the disease control group is unbalanced, which may bring certain predictive bias to the machine learning model. In addition, there is a significant imbalance in the gender distribution among the groups: all the subjects in the lupus group are female, while the healthy control group includes male subjects. Patients with rheumatoid arthritis, Sjogren’s syndrome and dermatomyositis are mainly female. Existing literature suggests that the core Raman characteristic peaks screened in this study have no obvious gender dependence. However, under the current small sample conditions, it is impossible to completely rule out the confounding interference caused by gender-related metabolic differences. Due to the limitation of sample size, this study did not stratify lupus patients based on clinical subtypes such as lupus nephritis and cutaneous lupus erythematosus. The efficacy of this Laman-artificial intelligence detection system in differentiating different lupus subtypes still needs to be verified.

Secondly, Raman spectroscopy can only reflect the relative intensity changes of biomolecular vibration signals within cells and cannot directly prove the causal relationship between metabolic alterations and the pathogenesis of lupus. The molecular attribution of characteristic peaks such as monosaccharides, phenylalanine, and nucleic acids in the study was all based on previous literature reports. However, metabolomics, transcriptomics, and cell function experiments are still needed to further verify the differential metabolites screened out by Raman fingerprinting.

Thirdly, the current entire testing process relies on high-precision laboratory confocal Raman microscopes, which have high equipment purchase and maintenance costs and are difficult to be directly popularized in primary medical institutions. This study did not include longitudinal follow-up data of lupus patients, and thus was unable to assess whether Raman signals from peripheral blood mononuclear cells could predict disease recurrence or the response effect to drug treatment.

In response to the above limitations, a multi-centre large cohort with a sample size of over 500 cases will be constructed subsequently. Age and gender of each group will be strictly matched, and clinical data related to SLEDAI (27) disease activity scores and organ involvement of patients will be collected simultaneously. Carry out mechanism verification by integrating multi-omics technologies and develop portable Raman detection devices to further promote the clinical application and transformation of this rapid lupus erythematosus detection technology.

## Data Availability

The original contributions presented in the study are included in the article/**Supplementary Material**. Further inquiries can be directed to the corresponding author.

## Supplementary material

Supplementary Figure 1 Hierarchical clustering heatmap of differential Raman spectral features across HC, SLE, and SS+RA+DM groups.

Supplementary Figure 2 Raman spectra of solvent blanks and process blanks showing negligible signals.
