# Supplementary material for: Research on a rapid detection method for systemic lupus erythematosus based on Raman spectroscopy
Source: Front Immunol. 2026 Aug 18;17:1806438. doi: 10.3389/fimmu.2026.1806438 (PMC13529956; doi:10.3389/fimmu.2026.1806438)
Supplement: Supplementary file 1 [file Table1.docx]

**supplemental figure S1**

**figure S1**:The hierarchical clustering heatmap demonstrates the variations in Raman spectral feature intensity among three study groups: Systemic Lupus Erythematosus (SLE) patients, disease control subjects (including Sjögren's syndrome [SS], rheumatoid arthritis [RA], and dermatomyositis [DM]), and healthy controls (HC). The colour scale indicates normalised Raman signal intensity, with blue denoting low intensity and red denoting high intensity. Rows correspond to individual Raman spectral features, labelled by feature identifiers reflecting specific molecular vibration patterns, while columns represent samples within each group. Both rows (features) and columns (samples) have undergone hierarchical clustering to visually display spectral similarities between groups.

**supplemental figure S2**
